# Supplementary material for: Immune modules to guide diagnosis and personalized treatment of inflammatory skin diseases
Source: Nat Commun. 2024 Dec 18;15:10688. doi: 10.1038/s41467-024-54559-6 (PMC11655867; doi:10.1038/s41467-024-54559-6)
Supplement: Supplementary file 1 — Supplementary Information [file 41467_2024_54559_MOESM1_ESM.pdf]

Inventory of Supplementary Information

Supplementary Figure 1. Representative clinical and histopathology images of sentinel and other inflammatory skin diseases.

Supplementary Figure 2. The full immune gene panel allows a certain degree of probe clustering by disease.

Supplementary Figure 3. Dissection of the myeloid signature into neutrophilic, macrophagic, and eosinophilic modules.

Supplementary Figure 4. RNA sequencing also classifies inflammatory skin diseases when using the module gene panel.

Supplementary Figure 5. Disease clustering and module expression are independent on the anatomical location of the disease and are stable over time.

Supplementary Figure 6. Differentially expressed genes in BP and DHR belong to the already defined immune modules.

Supplementary Figure 7. Representative clinical and histopathology images of non-responder patients.

Supplementary Figure 8. Count distribution per gene.

Supplementary Table 1. Significance level of modules

Supplementary Table 2. Clinical, histolopathology, and molecular diagnosis of 30 erythroderma patients

Supplementary Table 3. Clinical, histolopathology, and molecular diagnosis of 20 undetermined rashes

Supplementary Table 4. Comprehensive list of biopsies

Supplementary Table 5. Baseline characteristics of profiled patients

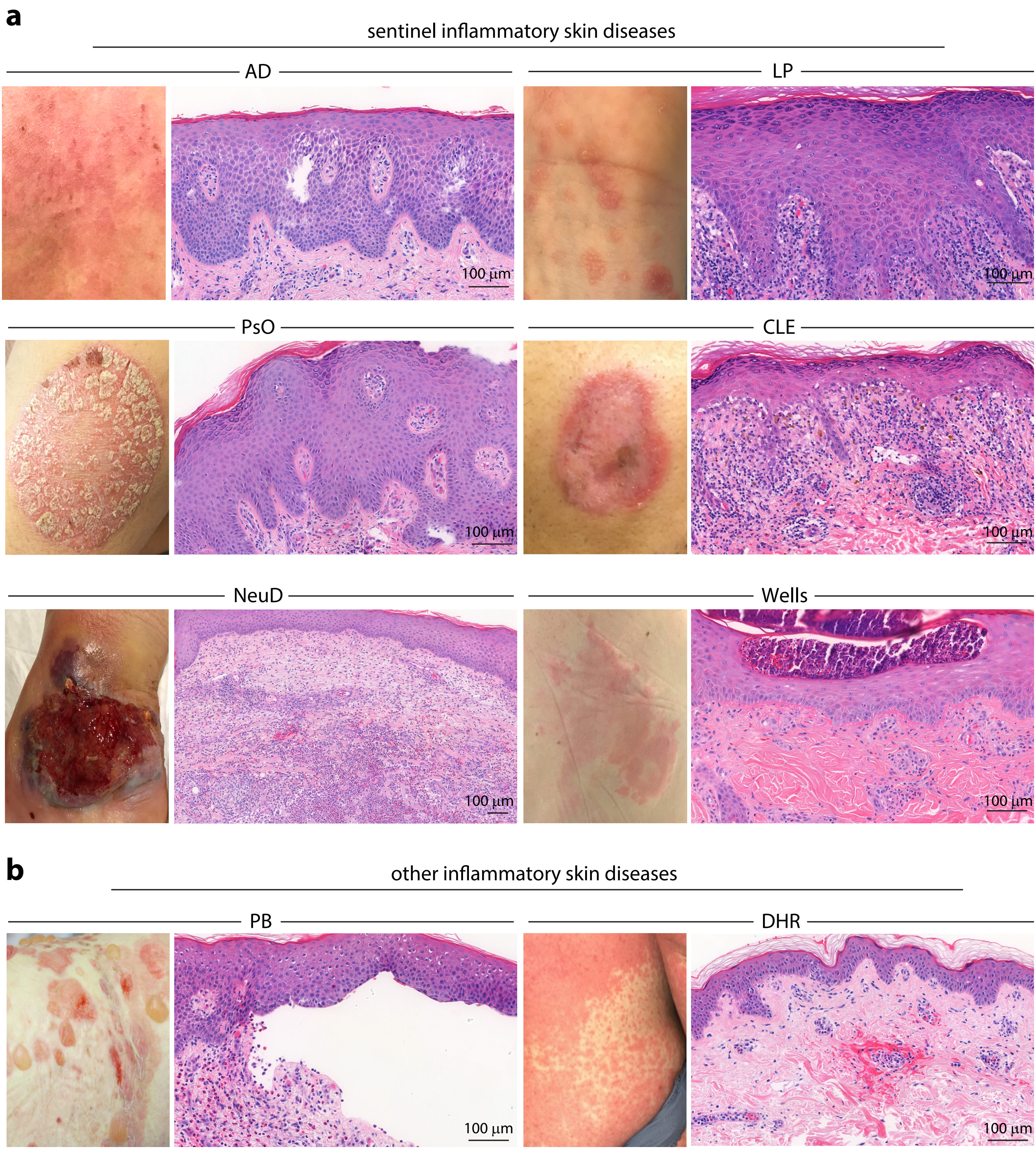

Supplementary Figure 1. Representative clinical and histopathology images of sentinel and other inflammatory skin diseases. Representative clinical (left) and histopathology (right) images of well-defined diseases referred to as "sentinels" including psoriasis (PsO, PsO_004), atopic dermatitis (AD, AD_008), neutrophilic diseases (NeuD, PG_001), lichen planus (LP, LP_011), cutaneous lupus erythematosus (CLE, CLE_010), and Wells syndrome patients (Wells, WELL_002) (**a**) and other inflammatory skin diseases including bullous pemphigoid (BP, BP_013) and maculopapular drug hypersensitivity reactions (DHR, DHR_002) (**b**). Scale bars are shown on histology images.

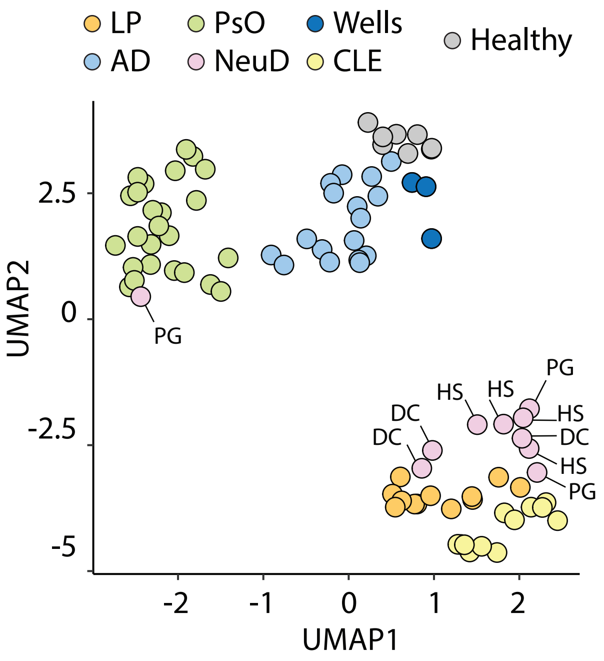

Supplementary Figure 2. The full immune gene panel allows a certain degree of probe clustering by disease.
UMAP projection of sentinel profiles based on the full gene panel (600 immune genes).
Diseases belonging to the group of neutrophilic dermatoses are specified.
HS, Hidradenitis suppurativa; PG, Pyoderma gangrenosum; DC, Dissecting cellulitis.

a

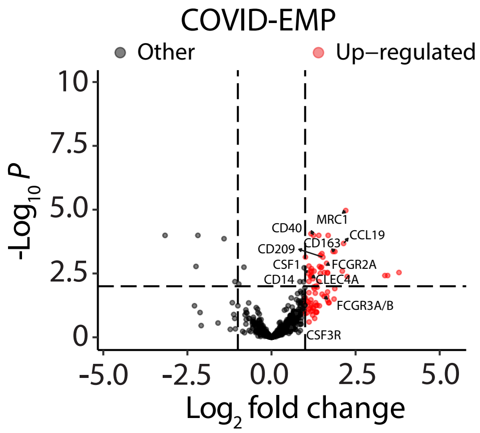

b

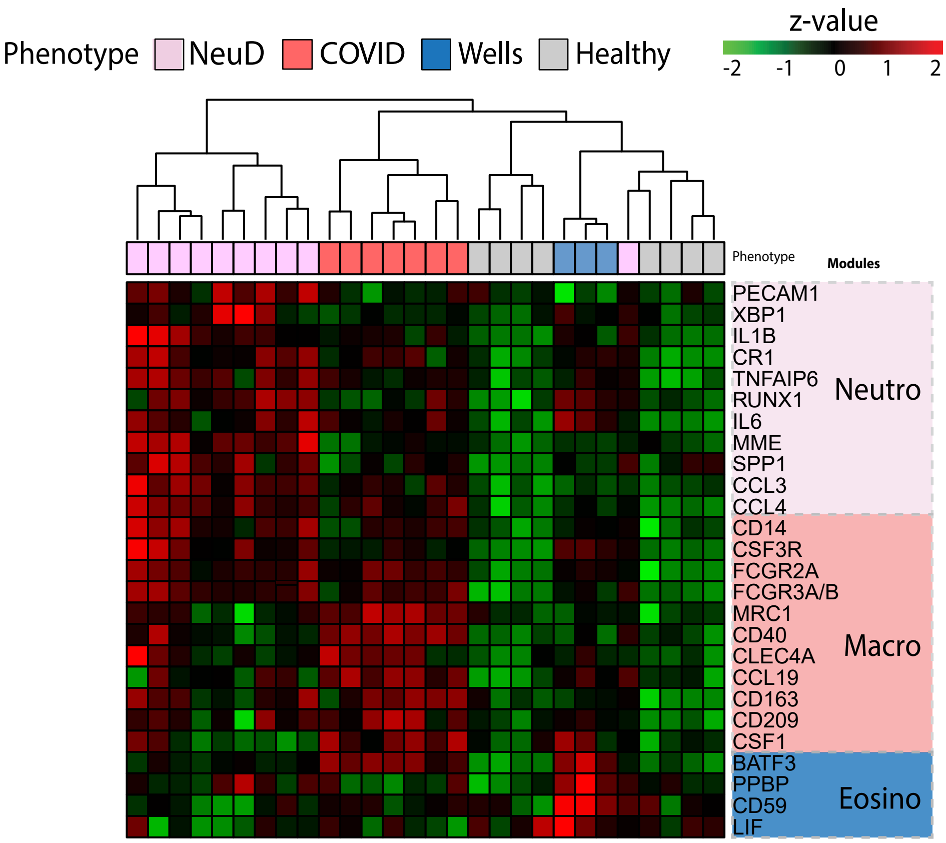

Supplementary Figure 3. Dissection of the myeloid signature into neutrophilic, macrophagic, and eosinophilic modules.
(**a**) Probes derived from COVID skin manifestations patients were profiled by Nanostring.
The expression levels for COVID were plotted against profiles of all other diseases to identify differentially expressed genes (DEG) that define the macrophagic module. Volcano plot depict DEGs, with dashed lines indicating the significance threshold defined as log2 fold change > 2 and p value < 0.01 derived from two-sided t -test.
(**b**) Heatmap of NeuD, COVID, and Wells probes showing disease-specific expression of the myeloid module divided into neutrophilic, macrophagic, and eosinophilic modules. The color gradient reflects expression levels given as z-scores.

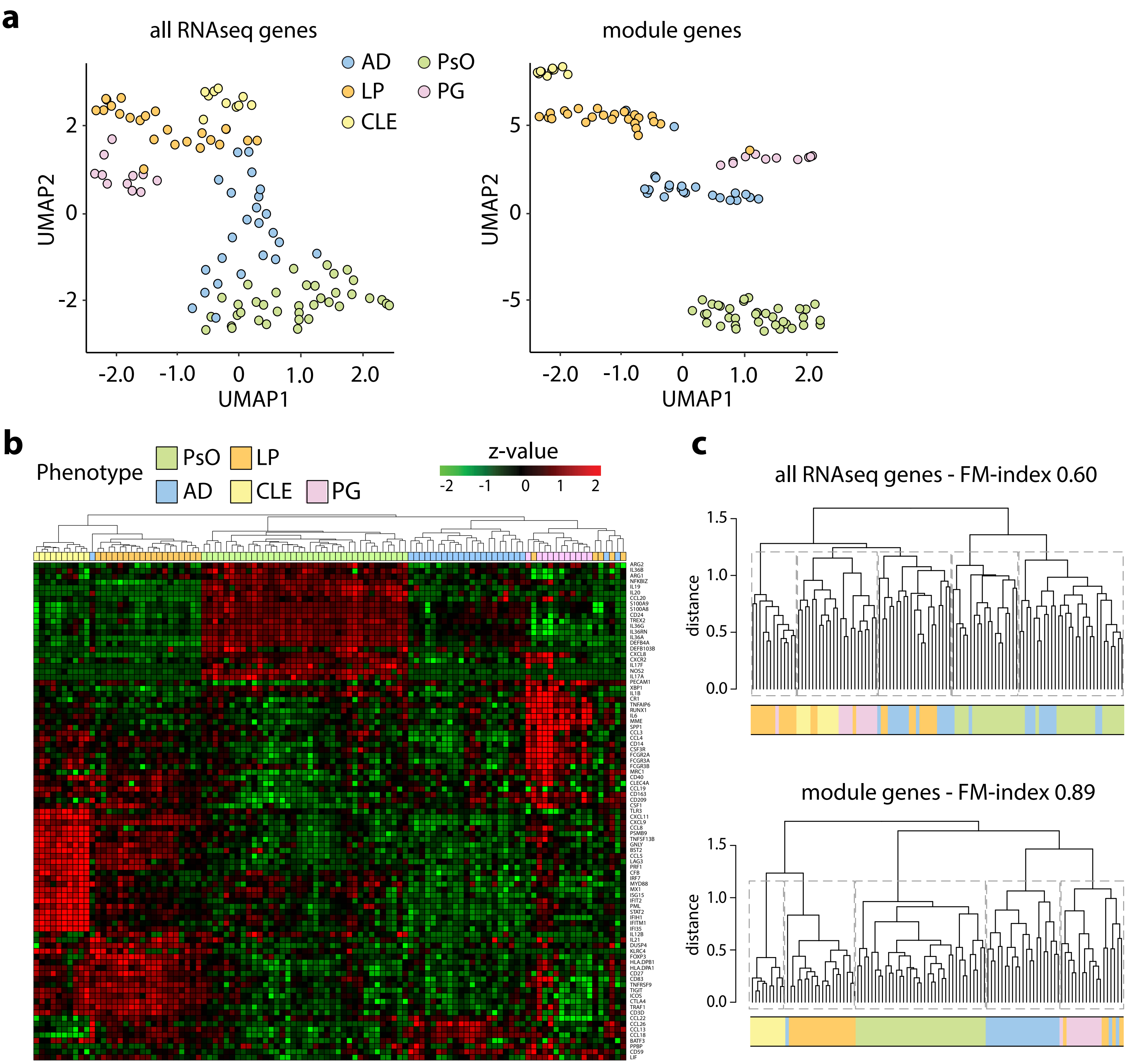

Supplementary Figure 4. RNA sequencing also classifies inflammatory skin diseases when using the module gene panel. (**a**) UMAP projection of RNA sequencing profiles in AD, PsO, CLE, LP, and PG patients using the full gene list (left) or the module gene panel (right). (**b**) Heatmap showing the RNAseq expression of module genes in AD, PsO, CLE, LP, and PG patients. The color gradient reflects expression levels given as z-scores. (**c**) Hierarchical clustering shown by dendrogram comparing the full list of genes with the module gene panel. Clustering accuracy is indicated by the Fowlkes–Mallows (FM) index.

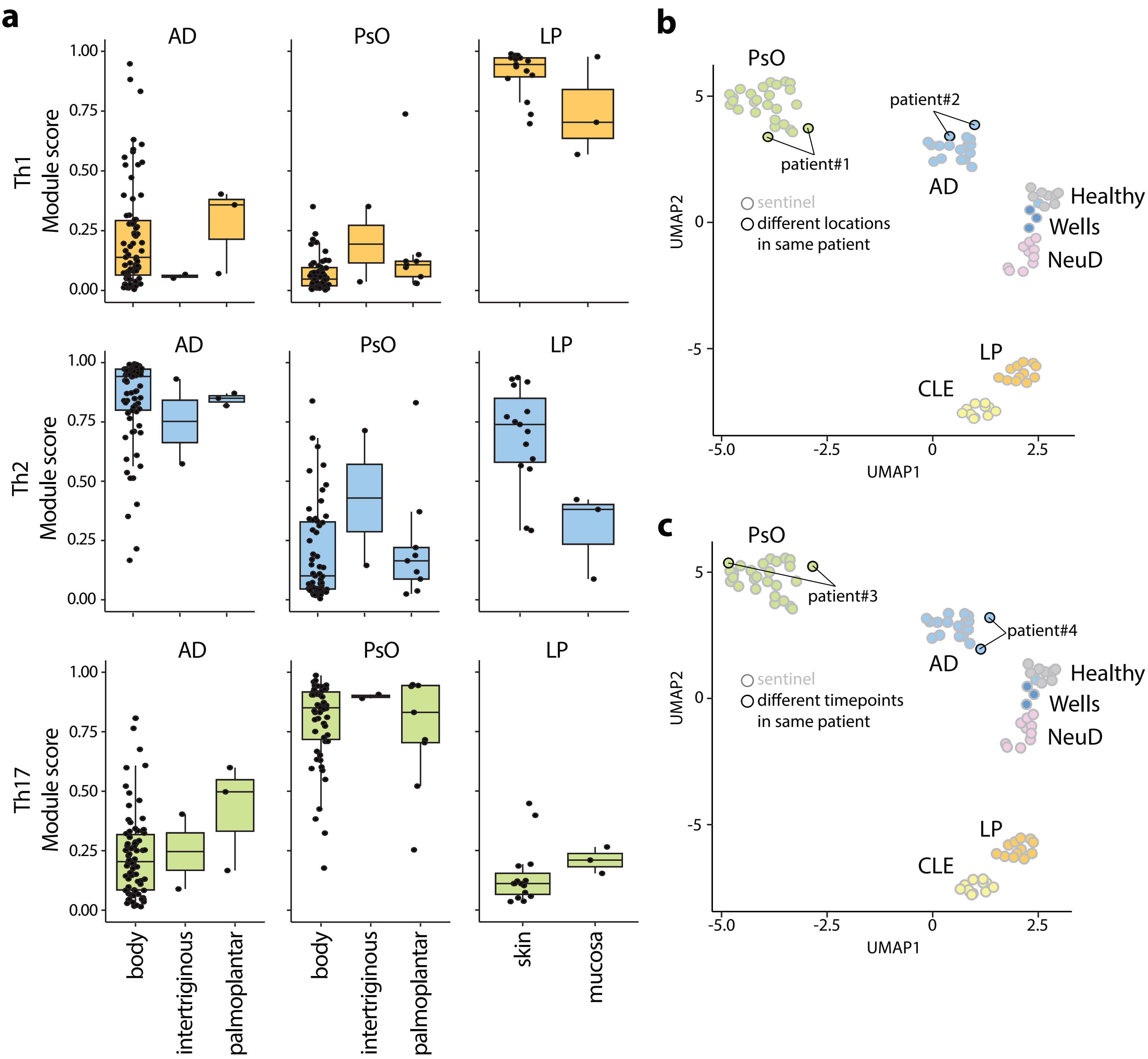

Supplementary Figure 5. Disease clustering and module expression are independent on the anatomical location of the disease and are stable over time.

(**a**) Box plots showing the normalized expression scores of Th1, Th2, and Th17 modules in AD, PsO, and LP biopsies of different body location. Each dot represents one test probe.

AD (n=74 body, 2 intertriginous, 3 palmoplantar), PsO (n=56 body, 2 intertriginous, 9 palmoplantar), LP (n=15 skin, 3 mucosa).

The central line of the box plot is the median. The box's edges are the lower (25th percentile) and upper quartiles (75th percentile).

Whiskers extend to data points within 1.5 times the interquartile range (IQR).

(**b**) UMAP projection of profiles from biopsies of the same patient (#1 and #2) taken at different locations (grey rings) and plotted against the established sentinel cartography (black rings).

(**c**) UMAP projection of profiles from biopsies of the same patient (#1 and #2) taken at different timepoints (grey rings) and plotted against the established sentinel cartography (black rings).

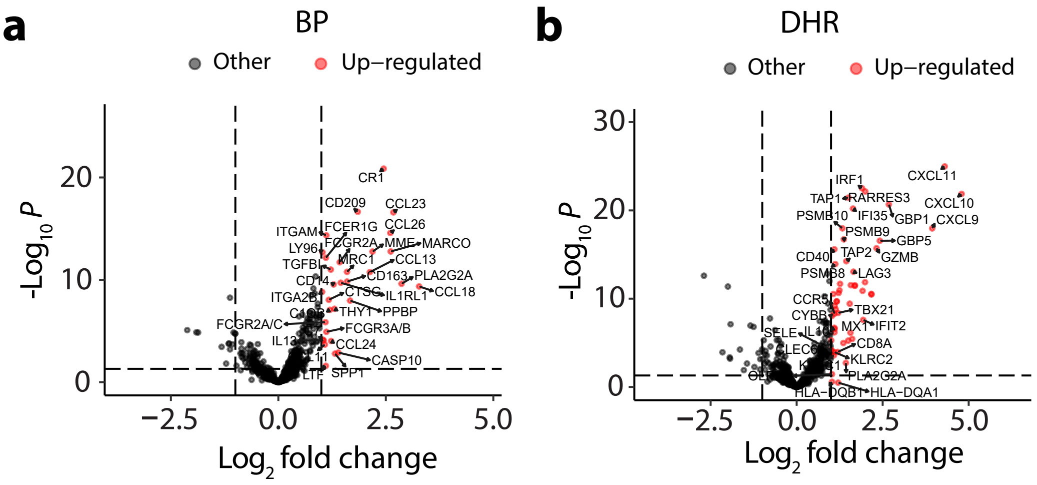

Supplementary Figure 6. Differentially expressed genes in BP and DHR belong to the already defined immune modules.
The expression levels for BP (**a**) and DHR (**b**) probes were plotted against profiles of all other diseases to identify differentially expressed genes (DEGs). Volcano plots depict DEGs, with dashed lines indicating the significance threshold defined as log2 fold change > 2 and p value < 0.01 derived from two-sided t-tests.
DEGs in BP probes are similar to the macrophagic module and DEGs in DHR to the IFN module.

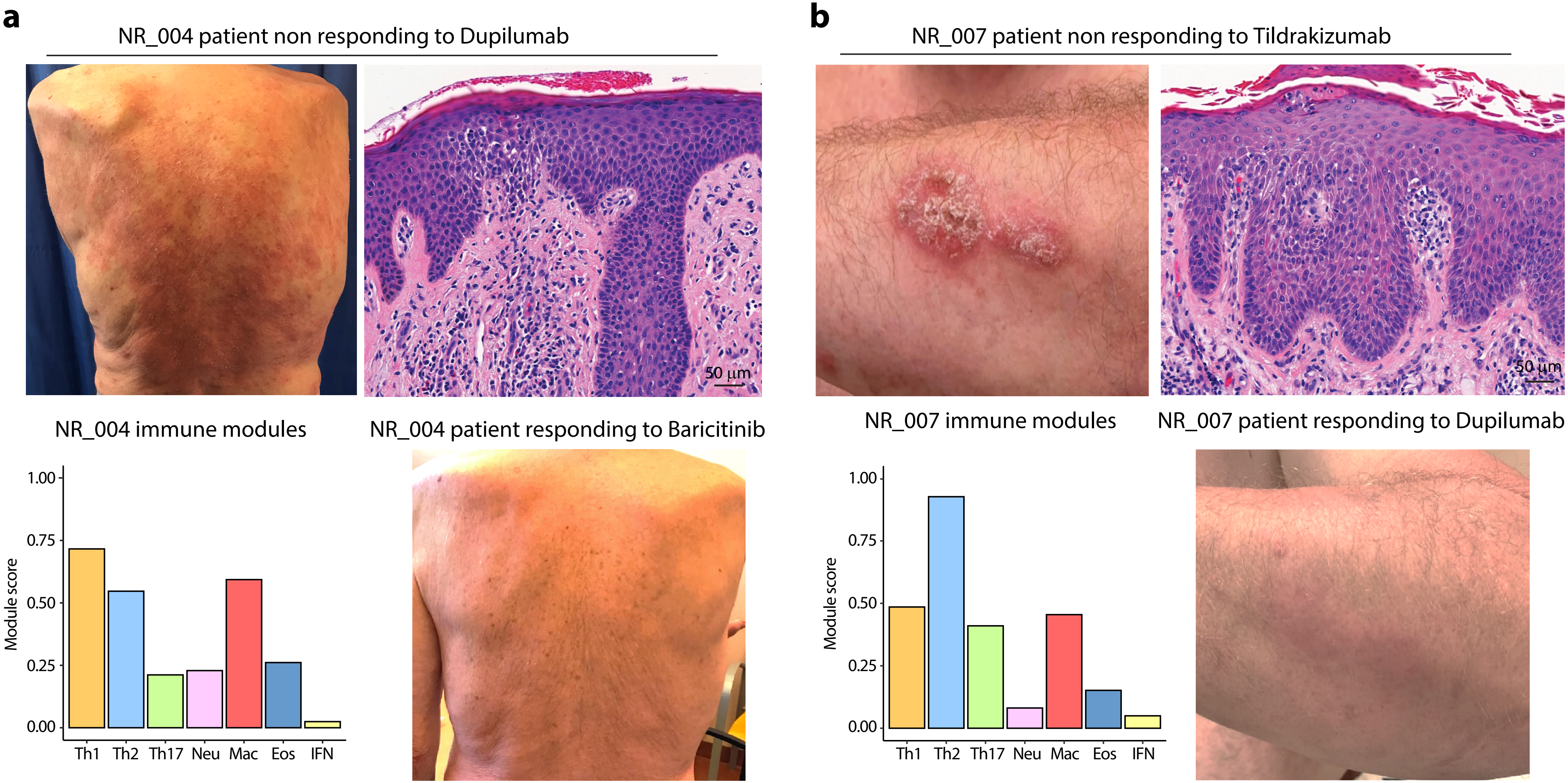

Supplementary Figure 7. Representative clinical and histopathology images of non-responder patients.
Clinical (top left) and histology (top right) images of initially diagnosed (**a**) atopic dermatitis (NR_004) and (**b**) psoriasis (NR_007) patients, who were resistant to targeted treatments and showed a mismatch immune profile at the molecular diagnosis (bottom left), responded to rematched treatment (bottom right). Scale bars are shown on histology images.

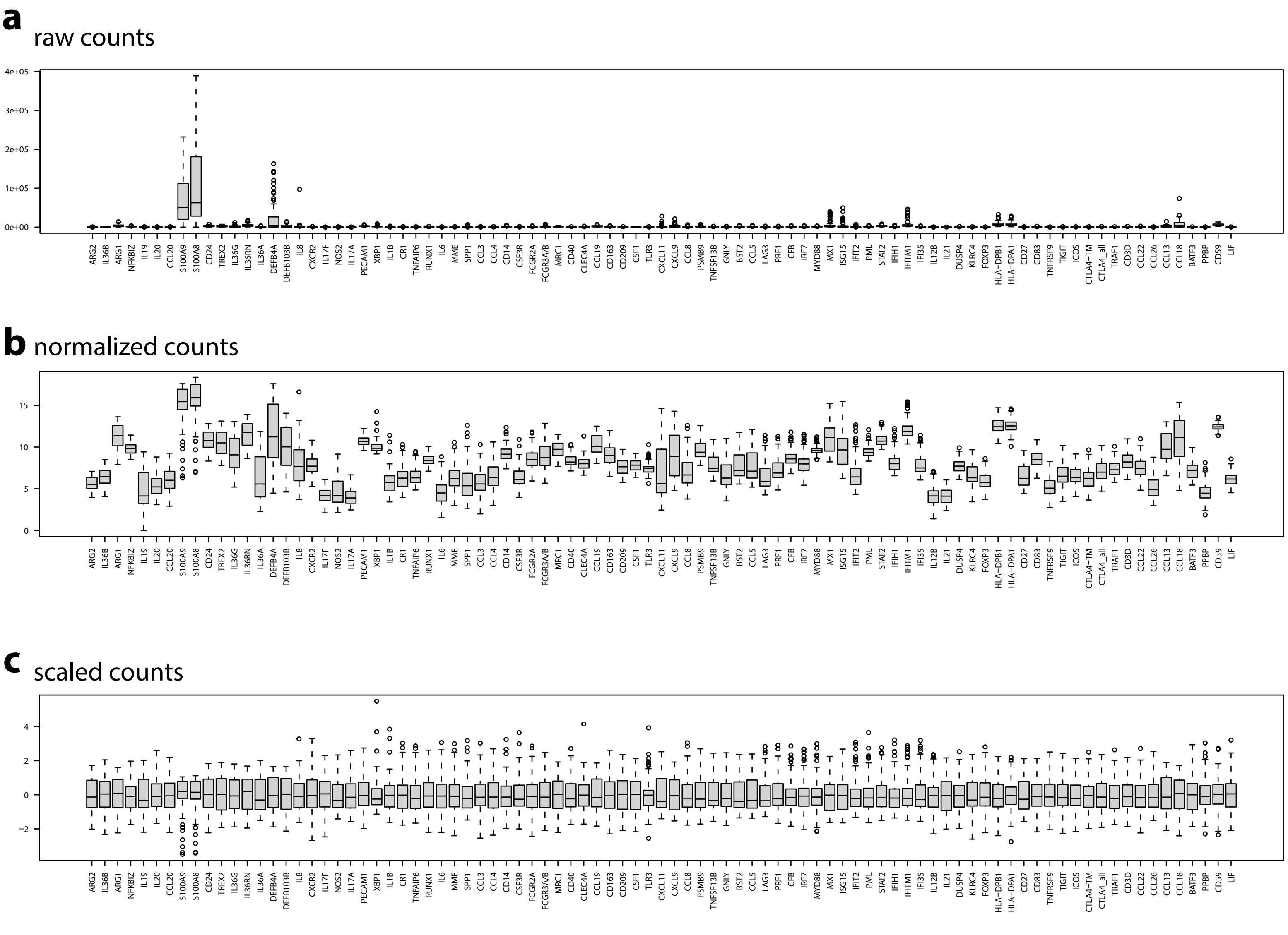

Supplementary Figure 8. Count distribution per gene.

Boxplots of raw counts (**a**), normalized counts (**b**), and scaled counts (**c**) of each module gene of the sentinel probes (n=108).

The center of the box plot is marked by the median, a line splitting the box in half.

The box's edges are the lower (25th percentile) and upper quartiles (75th percentile).

Whiskers extend to data points within 1.5 times the interquartile range (IQR). Points outside this range are outliers, shown separately.

Supplementary Table 1. Significance level of modules

Group	Phenotype	Th2	Th1	IFN	Neutro	Th17	Eosino	Macro
sentinel	AD	NA	3.33E-09	3.33E-09	3.33E-09	6.65E-09	3.33E-09	6.65E-09
sentinel	LP	3.71E-04	NA	7.40E-07	7.40E-07	7.40E-07	7.40E-07	4.96E-05
sentinel	Lupus	2.96E-06	2.91E-03	NA	1.41E-05	7.40E-07	7.40E-07	4.96E-05
sentinel	ND	1.08E-05	4.33E-05	1.08E-05	NA	1.08E-05	1.08E-05	6.30E-02
sentinel	PSO	1.11E-13	1.58E-14	1.58E-14	3.16E-14	NA	1.58E-14	1.58E-14
sentinel	Wells	1.00E-01	1.00E-01	1.00E-01	1.00E-01	1.00E-01	NA	2.00E-01
test	AD	NA	1.29E-08	1.29E-08	2.58E-08	1.29E-08	5.16E-08	9.03E-08
test	LP	6.49E-02	NA	2.16E-03	4.11E-02	2.16E-03	2.16E-03	1.32E-01
test	Lupus	5.83E-04	4.08E-03	NA	2.33E-03	5.83E-04	5.83E-03	4.08E-03
test	ND	1.59E-02	1.59E-02	7.94E-03	NA	3.17E-02	3.17E-02	8.41E-01
test	PSO	4.11E-05	4.11E-05	4.11E-05	4.11E-05	NA	4.11E-05	4.11E-05

p-values from wilcoxon rank-sum two-sided test between mean module of each phenotype(row) versus the others (column)

Supplementary Table 2. Clinical, histolopathology, and molecular diagnosis of 30 erythroderma patients

Patient ID	Age	History of PsO, AD, and suspected drug	Initial Clinical Dx	Histopathology Dx (%)			Elements supporting Dx	Final Dx (long term evolution, response to treatment)	Molecular Dx (clustering/dominant module)
				PsO	AD	DHR			
Patient#1	89	-	AD vs DHR	13.3	50.0	36.7	Response to dupilumab	AD	AD (Th2)
Patient#2	61	-	AD vs DHR	0.0	60.0	40.0	Relapse with eczema lesions	AD	AD (Th2)
Patient#3	50	AD	AD	0.0	100.0	0.0	History of AD; Response to dupilumab	AD	AD (Th2)
Patient#4	79	-	PsO vs AD	23.3	43.3	33.3		AD	AD (Th2)
Patient#5	79	Torasemid	AD vs DHR	0.0	7.0	93.0	↑IgE ; persistence despite drug discontinuation	AD	AD (Th2)
Patient#6	79	-	PsO vs AD vs DHR	0.0	93.0	7.0	Relapse with eczema lesions	AD	AD (Th2)
Patient#7	71	AD	AD vs DHR	0.0	36.7	63.3	History of AD; ↑IgE	AD	AD (Th2)
Patient#8	68	-	DHR/AD	0.0	100.0	0.0	Histopathology; Response to dupilumab	AD	AD (Th2)
Patient#9	87	-	DHR/PB/PsO	0.0	100.0	0.0	Histopathology; Response to dupilumab	AD	AD (Th2)
Patient#10	56	-	AD/DHR	0.0	100.0	0.0	Histopathology; ↑IgE; Response to dupilumab	AD	AD (Th2)
Patient#11	70	AD	AD/DHR	0.0	100.0	0.0	Histopathology; ↑IgE; History of AD	AD	AD (Th2)
Patient#12	28	AD	AD	0.0	100.0	0.0	Histopathology; ↑IgE; History of AD	AD	AD (Th2)
Patient#13	71	AD	AD/DHR	0.0	100.0	0.0	Histopathology; History of AD	AD	AD (Th2)
Patient#14	37	AD	AD	0.0	100.0	0.0	Histopathology; History of AD	AD	AD (Th2)
Patient#15	81	-	AD/PsO	0.0	100.0	0.0	Histopathology; ↑IgE	AD	AD (Th2)
Patient#16	54	-	AD	0.0	100.0	0.0	Histopathology; ↑IgE	AD	AD (Th2)
Patient#17	83	AD	AD/PB	0.0	100.0	0.0	Histopathology; ↑IgE; History of AD	AD	AD (Th2)
Patient#18	31	Minocycline	DHR	0.0	0.0	100.0	Histopathology	DHR	DHR
Patient#19	62	Vancomycine	AD vs DHR	26.7	6.7	66.7	No relapse after drug discontinuation	DHR	DHR (Th1+Neutro+IFN)
Patient#20	59	Clarithromycine, Céfuroxime	AD vs DHR	0.0	33.3	66.7	No relapse after drug discontinuation	DHR	DHR (Th1+Neutro+IFN)
Patient#21	71	Carbamazepine	AD vs DHR	0.0	33.3	66.7	No relapse after drug discontinuation	DHR	DHR (Th1+Neutro+IFN)
Patient#22	75	-	PsO vs AD	90.0	10.0	0.0	Relapse with psoriasis lesions	PsO	PsO (Th17)
Patient#23	66	PsO	PsO	60.0	33.3	7.0	History of PsO	PsO	PsO (Th17)
Patient#24	35	PsO	PsO	100.0	0.0	0.0	Histopathology; Response to Anti-IL-36; History of PsO	PsO	PsO (Th17)
Patient#25	67	PsO	PsO	100.0	0.0	0.0	Histopathology; Response to Anti-IL-23; History of PsO	PsO	PsO (Th17)
Patient#26	79	PsO	PsO	100.0	0.0	0.0	Histopathology; Response to Anti-IL-17A; History of PsO	PsO	PsO (Th17)
Patient#27	68	PsO	DHR/PsO	100.0	0.0	0.0	Histopathology; History of PsO	PsO	PsO (Th17)
Patient#28	80	PsO	PsO	100.0	0.0	0.0	Histopathology; History of PsO	PsO	PsO (Th17)
Patient#29	76	-	AD	100.0	0.0	0.0	Histopathology	PsO	PsO (Th17)
Patient#30	83	PsO	PsO	100.0	0.0	0.0	Histopathology; History of PsO	PsO	PsO (Th17)

Supplementary Table 3. Clinical, histolopathology, and molecular diagnosis of 20 undetermined rashes

Patient ID	Age	Personal Hx of PsO, AD	Clinical Dx	Histolopathology Dx	Molecular Dx	Response to targeted treamtment
Patient#1	23	-	AD/PsO	AD/PsO	PsO (Th17)	Anti-IL-17A
Patient#2	77	-	AD/DHR	AD/DHR	AD (Th2)	Anti-IL-4R
Patient#3	75	PsO	AD/PsO	AD/PsO	PsO (Th17)	Anti-IL-23
Patient#4	39	Atopy	AD/PsO	AD/PsO	PsO (Th17)	Anti-IL-23
Patient#5	85	-	AD/PsO	AD/PsO	PsO (Th17)	Anti-IL-23
Patient#6	75	-	AD/PsO	AD/PsO	AD (Th2)	Anti-IL-4R
Patient#7	92	-	AD/PB/DHR	AD/LP	LP (Th1)	
Patient#8	50	IgE ↑	AD/PsO	AD/DHR	AD (Th2)	
Patient#9	37	PsO	AD/PsO	AD/PsO	PsO (Th17)	
Patient#10	20	PsO	AD/PsO	AD/PsO	PsO (Th17)	
Patient#11	40	-	AD/PsO	AD/PsO	PsO (Th17)	
Patient#12	32	PsO	AD/PsO	AD/PsO	PsO (Th17)	
Patient#13	95	-	AD/CLE/PsO/DHR/PB	AD/DHR	AD (Th2)	
Patient#14	69	Atopy	AD/PsO	AD/LP	LP (Th1)	
Patient#15	89	LP	AD/PB /LP	AD/PB	AD (Th2)	
Patient#16	62	-	AD/PsO/CLE	AD/PsO	PsO (Th17)	
Patient#17	58	PsO	AD/PsO	AD/PsO	AD (Th2)	
Patient#18	65	Atopy	AD/DHR	AD/DHR	PsO (Th17)	
Patient#19	78	-	AD/PsO	AD/DHR	AD (Th2)	
Patient#20	84	-	AD/PsO	AD/PsO	AD (Th2)	
Patient#21	44	-	AD/BP/DHR	AD/BP/DHR	BP (Th2, macro)	

Supplementary Table 4. Comprehensive list of biopsies

Code	Disease	Sentinel	Test	pre.treatment	post.treatment	Location	Age	Gender
AD_001	Atopic Dermatitis	√	-	-	-	Extremities	29	M
AD_002	Atopic Dermatitis	√	-	-	-	Trunk	45	M
AD_003	Atopic Dermatitis	-	√	√	-	Trunk	48	M
AD_004	Atopic Dermatitis	√	-	-	-	Extremities	24	F
AD_005	Atopic Dermatitis	-	√	√	-	Trunk	35	M
AD_006	Atopic Dermatitis	√	-	-	-	Trunk	41	M
AD_007	Atopic Dermatitis	√	-	-	-	Trunk	24	F
AD_008	Atopic Dermatitis	√	-	√	-	Extremities	54	M
AD_009	Atopic Dermatitis	-	√	√	-	Extremities	63	M
AD_010	Atopic Dermatitis	-	√	-	-	Extremities	46	F
AD_011	Atopic Dermatitis	-	-	√	-	Extremities	74	M
AD_012	Atopic Dermatitis	-	-	√	-	Trunk	68	F
AD_013	Atopic Dermatitis	-	-	√	-	Trunk	31	M
AD_014	Atopic Dermatitis	-	-	√	-	Extremities	44	F
AD_015	Atopic Dermatitis	√	-	-	-	Extremities	31	F
AD_016	Atopic Dermatitis	√	-	√	-	Extremities	32	M
AD_017	Atopic Dermatitis	√	-	√	-	Extremities	30	M
AD_018	Atopic Dermatitis	√	-	-	-	Trunk	83	M
AD_019	Atopic Dermatitis	-	√	√	-	Trunk	50	M
AD_020	Atopic Dermatitis	-	√	√	-	Trunk	29	M
AD_021	Atopic Dermatitis	-	-	√	-	Trunk	83	M
AD_022	Atopic Dermatitis	-	-	√	-	Extremities	66	M
AD_023	Atopic Dermatitis	-	√	√	-	Trunk	52	M
AD_024	Atopic Dermatitis	-	√	√	-	Trunk	37	M
AD_025	Atopic Dermatitis	-	-	√	-	Trunk	70	M
AD_026	Atopic Dermatitis	-	-	√	-	Trunk	80	M
AD_027	Atopic Dermatitis	-	-	√	-	Extremities	83	F
AD_028	Atopic Dermatitis	-	-	√	-	Extremities	79	M
AD_029	Atopic Dermatitis	-	-	√	-	Extremities	49	M
AD_030	Atopic Dermatitis	-	-	√	-	Trunk	61	M
AD_031	Atopic Dermatitis	-	-	√	-	Trunk	62	M
AD_032	Atopic Dermatitis	-	-	√	-	Extremities	83	M
AD_033	Atopic Dermatitis	-	√	-	-	Extremities	40	M
AD_034	Atopic Dermatitis	-	√	-	-	Extremities	49	M
AD_035	Atopic Dermatitis	√	-	-	-	Extremities	41	M
AD_036	Atopic Dermatitis	√	-	-	-	Extremities	28	F
AD_037	Atopic Dermatitis	-	-	√	-	Trunk	61	M
AD_038	Atopic Dermatitis	-	-	√	-	Trunk	79	M
AD_039	Atopic Dermatitis	√	-	-	-	Extremities	34	M
AD_040	Atopic Dermatitis	-	-	√	-	Trunk	84	F
AD_041	Atopic Dermatitis	-	-	√	-	Extremities	58	M
AD_042	Atopic Dermatitis	√	-	√	-	Extremities	48	M
AD_043	Atopic Dermatitis	√	-	-	-	Trunk	28	M
AD_044	Atopic Dermatitis	-	√	-	-	Head and neck	33	M
AD_045	Atopic Dermatitis	-	√	√	-	Extremities	25	F
AD_046	Atopic Dermatitis	-	√	√	-	Extremities	34	M
AD_047	Atopic Dermatitis	-	√	-	-	Extremities	47	F
AD_048	Atopic Dermatitis	-	-	√	-	Trunk	62	M
AD_049	Atopic Dermatitis	-	-	√	-	Extremities	67	M
AD_050	Atopic Dermatitis	√	-	√	-	Trunk	63	M
AD_051	Atopic Dermatitis	-	√	√	-	Extremities	26	M
AD_052	Atopic Dermatitis	-	-	-	-	Trunk	67	F
AD_053	Atopic Dermatitis	-	-	-	-	Extremities	67	F
AD_054	Atopic Dermatitis	-	-	-	-	Extremities	77	M
AD_055	Atopic Dermatitis	-	-	-	-	Intertriginous	93	F
AD_056	Atopic Dermatitis	-	-	-	-	Palmoplantar	91	M
AD_057	Atopic Dermatitis	-	-	-	-	Palmoplantar	75	F
AD_058	Atopic Dermatitis	-	-	-	-	Palmoplantar	47	M
BP_001	Bullous pemphigoid	-	-	√	-	Extremities	78	F
BP_002	Bullous pemphigoid	-	-	√	-	Extremities	56	F
BP_003	Bullous pemphigoid	√	-	-	-	Extremities	86	M
BP_004	Bullous pemphigoid	√	-	-	-	Trunk	83	M
BP_005	Bullous pemphigoid	√	-	-	-	Trunk	91	M
BP_006	Bullous pemphigoid	√	-	√	-	Trunk	77	F
BP_007	Bullous pemphigoid	-	-	√	-	Trunk	84	M
BP_008	Bullous pemphigoid	√	-	-	-	Trunk	68	M
BP_009	Bullous pemphigoid	√	-	-	-	Extremities	77	F
BP_010	Bullous pemphigoid	√	-	-	-	Extremities	71	F
BP_011	Bullous pemphigoid	√	-	-	-	Extremities	80	M
BP_012	Bullous pemphigoid	√	-	-	-	Extremities	93	M
BP_013	Bullous pemphigoid	√	-	-	-	Extremities	83	F
BP_014	Bullous pemphigoid	√	-	-	-	Extremities	89	M
BP_015	Bullous pemphigoid	√	-	-	-	Extremities	81	M
BP_016	Bullous pemphigoid	-	-	√	-	Extremities	87	M
BP_017	Bullous pemphigoid	-	-	√	-	Extremities	75	F
CLE_001	Cutaneous lupus	√	-	-	-	Head and neck	79	M
CLE_002	Cutaneous lupus	-	√	-	-	Extremities	71	M
CLE_003	Cutaneous lupus	-	√	-	-	Extremities	76	F
CLE_004	Cutaneous lupus	-	√	-	-	Extremities	88	F
CLE_005	Cutaneous lupus	√	-	-	-	Trunk	53	F
CLE_006	Cutaneous lupus	√	-	-	-	Head and neck	28	F
CLE_007	Cutaneous lupus	√	-	-	-	Head and neck	54	M
CLE_008	Cutaneous lupus	√	-	-	-	Extremities	80	F
CLE_009	Cutaneous lupus	-	√	-	-	Head and neck	39	M
CLE_010	Cutaneous lupus	√	-	-	-	Head and neck	42	M
CLE_011	Cutaneous lupus	√	-	-	-	Head and neck	42	F
CLE_012	Cutaneous lupus	√	-	-	-	Head and neck	48	F
CLE_013	Cutaneous lupus	-	√	-	-	Trunk	58	F
CLE_014	Cutaneous lupus	√	-	-	-	Head and neck	65	F
CLE_015	Cutaneous lupus	√	-	-	-	Head and neck	48	M
CLE_016	Cutaneous lupus	√	-	-	-	Head and neck	48	F
CLE_017	Cutaneous lupus	√	-	-	-	Extremities	38	M

CLE_018	Cutaneous lupus	-	√	-	-	Extremities	51	F
CLE_019	Cutaneous lupus	-	√	-	-	Head and neck	63	F
DC_001	Dissecans cellulitis	√	-	-	-	Head and neck	29	M
DC_002	Dissecans cellulitis	√	-	-	-	Head and neck	27	M
DC_003	Dissecans cellulitis	√	-	-	-	Head and neck	42	M
DHR_001	Drug hypersensitivity reaction	√	-	-	-	Trunk	94	F
DHR_002	Drug hypersensitivity reaction	√	-	-	-	Trunk	64	F
DHR_003	Drug hypersensitivity reaction	√	-	-	-	Extremities	60	F
DHR_004	Drug hypersensitivity reaction	√	-	-	-	Extremities	60	M
DHR_005	Drug hypersensitivity reaction	√	-	-	-	Extremities	38	F
DHR_006	Drug hypersensitivity reaction	√	-	-	-	Extremities	79	M
DHR_007	Drug hypersensitivity reaction	√	-	-	-	Trunk	46	M
DHR_008	Drug hypersensitivity reaction	√	-	-	-	Extremities	21	M
DHR_009	Drug hypersensitivity reaction	√	-	-	-	Trunk	65	F
DHR_010	Drug hypersensitivity reaction	√	-	-	-	Trunk	42	M
Eryth_001	Erythroderma	-	-	-	-	Extremities	60	F
Eryth_002	Erythroderma	-	-	-	-	Extremities	78	M
Eryth_003	Erythroderma	-	-	√	-	Extremities	49	M
Eryth_004	Erythroderma	-	-	-	-	Extremities	70	M
Eryth_005	Erythroderma	-	-	-	-	Trunk	78	M
Eryth_006	Erythroderma	-	-	-	-	Extremities	88	M
Eryth_007	Erythroderma	-	-	-	-	Trunk	78	M
Eryth_008	Erythroderma	-	-	√	-	Extremities	56	F
Eryth_009	Erythroderma	-	-	-	-	Extremities	70	M
Eryth_010	Erythroderma	-	-	-	-	Trunk	72	M
Eryth_011	Erythroderma	-	-	-	-	Extremities	54	M
Eryth_012	Erythroderma	-	-	-	-	Extremities	76	F
Eryth_013	Erythroderma	-	-	-	-	Extremities	37	F
Eryth_014	Erythroderma	-	-	-	-	Extremities	80	M
Eryth_015	Erythroderma	-	-	-	-	Extremities	83	M
Eryth_016	Erythroderma	-	-	-	-	Trunk	67	M
Eryth_017	Erythroderma	-	-	-	-	Extremities	67	M
Eryth_018	Erythroderma	-	-	-	-	Extremities	65	M
Eryth_019	Erythroderma	-	-	-	-	Trunk	74	M
Eryth_020	Erythroderma	-	-	-	-	Trunk	79	F
Eryth_021	Erythroderma	-	-	-	-	Trunk	28	M
Eryth_022	Erythroderma	-	-	-	-	Trunk	89	M
Eryth_023	Erythroderma	-	-	-	-	Extremities	35	M
Eryth_024	Erythroderma	-	-	-	-	Trunk	66	M
Eryth_025	Erythroderma	-	-	√	-	Extremities	82	F
Eryth_026	Erythroderma	-	-	-	-	Trunk	78	F
Eryth_027	Erythroderma	-	-	-	-	Extremities	61	M
Eryth_028	Erythroderma	-	-	-	-	Trunk	58	M
Eryth_029	Erythroderma	-	-	-	-	Trunk	70	M
Eryth_030	Erythroderma	-	-	-	-	Trunk	31	M
HD_001	Healthy skin	√	-	-	-			
HD_002	Healthy skin	√	-	-	-			
HD_003	Healthy skin	√	-	-	-	Extremities	45	M
HD_004	Healthy skin	√	-	-	-	Head and neck	84	M
HD_005	Healthy skin	√	-	-	-	Extremities	68	M
HD_006	Healthy skin	√	-	-	-	Trunk	57	M
HD_007	Healthy skin	√	-	-	-	Extremities	23	M
HD_008	Healthy skin	√	-	-	-	Head and neck	39	M
HS_001	Hidradenitis suppurativa	√	-	-	-	Trunk	60	M
HS_002	Hidradenitis suppurativa	√	-	-	-	Trunk	31	M
HS_003	Hidradenitis suppurativa	√	-	-	-	Head and neck	29	M
HS_004	Hidradenitis suppurativa	√	-	-	-	Intertriginous	42	M
HS_005	Hidradenitis suppurativa	-	√	-	-	Extremities	36	M
LP_001	Lichen planus	√	-	-	-	Trunk	40	M
LP_002	Lichen planus	-	√	-	-	Extremities	68	M
LP_003	Lichen planus	√	-	-	-	Extremities	35	M
LP_004	Lichen planus	√	-	-	-	Extremities	75	F
LP_005	Lichen planus	√	-	-	-	Trunk	58	M
LP_006	Lichen planus	√	-	-	-	Extremities	56	F
LP_007	Lichen planus	√	-	-	-	Extremities	52	M
LP_008	Lichen planus	√	-	-	-	Extremities	38	F
LP_009	Lichen planus	√	-	-	-	Extremities	38	F
LP_010	Lichen planus	√	-	-	-	Extremities	48	F
LP_011	Lichen planus	√	-	-	-	Extremities	34	F
LP_012	Lichen planus	√	-	-	-	Intertriginous	62	M
LP_013	Lichen planus	√	-	-	-	Trunk	41	F
LP_014	Lichen planus	-	√	-	-	Extremities	38	M
LP_015	Lichen planus	-	√	√	-	Buccal	65	M
LP_016	Lichen planus	-	√	-	-	Extremities	26	M
LP_017	Lichen planus	-	√	-	-	Buccal	52	F
LP_018	Lichen planus	-	√	-	-	Buccal	33	M
NR_001	Non responder	-	-	√	√	Extremities	67	M
NR_002	Non responder	-	-	-	√	Trunk	66	M
NR_003	Non responder	-	-	-	√	Extremities	66	M
NR_004	Non responder	-	-	-	√	Trunk	79	M
NR_005	Non responder	-	-	-	√	Trunk	33	M
NR_006	Non responder	-	-	√	√	Extremities	88	F
NR_007	Non responder	-	-	-	√	Extremities	65	M
NR_008	Non responder	-	-	√	√	Trunk	66	M
NR_009	Non responder	-	-	-	√	Head and neck	61	M
NR_010	Non responder	-	-	-	√	Extremities	59	M
NR_011	Non responder	-	-	-	√		58	M
NR_012	Non responder	-	-	-	√		56	M
NR_013	Non responder	-	-	-	√		65	M
NR_014	Non responder	-	-	-	√	Extremities	47	F
NR_015	Non responder	-	-	-	√	Palmoplantar	55	M
NR_016	Non responder	-	-	-	√	Extremities	59	M
NR_017	Non responder	-	-	-	√	Head and neck	18	F
PG_001	Pyoderma gangrenosum	√	-	-	-	Extremities	88	F

PG_002	Pyoderma gangrenosum	-	√	-	-	Trunk	19	F
PG_003	Pyoderma gangrenosum	√	-	-	-	Extremities	77	M
PG_004	Pyoderma gangrenosum	√	-	-	-	Genital	61	M
PG_005	Pyoderma gangrenosum	-	√	-	-	Extremities	46	M
PsO_001	Psoriasis	-	√	√	-	Trunk	37	F
PsO_002	Psoriasis	√	-	-	-	Extremities	34	M
PsO_003	Psoriasis	√	-	-	-	Trunk	67	M
PsO_004	Psoriasis	√	-	-	-	Extremities	23	M
PsO_005	Psoriasis	√	-	√	-	Trunk	36	M
PsO_006	Psoriasis	√	-	-	-	Extremities	45	M
PsO_007	Psoriasis	√	-	√	-	Trunk	40	M
PsO_008	Psoriasis	√	-	√	-	Extremities	50	F
PsO_009	Psoriasis	√	-	√	-	Extremities	64	M
PsO_010	Psoriasis	√	-	√	-	Extremities	58	M
PsO_011	Psoriasis	-	√	√	-	Extremities	43	M
PsO_012	Psoriasis	√	-	√	-	Extremities	43	M
PsO_013	Psoriasis	√	-	-	-	Extremities	75	M
PsO_014	Psoriasis	√	-	-	-	Extremities	30	M
PsO_015	Psoriasis	√	-	√	-	Extremities	45	M
PsO_016	Psoriasis	-	√	-	-	Extremities	61	F
PsO_017	Psoriasis	√	-	-	-	Extremities	50	M
PsO_018	Psoriasis	√	-	-	-	Trunk	68	M
PsO_019	Psoriasis	√	-	-	-	Trunk	49	F
PsO_020	Psoriasis	-	-	√	-	Palmoplantar	57	F
PsO_021	Psoriasis	√	-	-	-	Extremities	80	M
PsO_022	Psoriasis	√	-	√	-	Trunk	57	F
PsO_023	Psoriasis	√	-	-	-	Extremities	46	M
PsO_024	Psoriasis	√	-	-	-	Extremities	46	M
PsO_025	Psoriasis	√	-	√	-	Extremities	66	M
PsO_026	Psoriasis	√	-	-	-	Extremities	72	M
PsO_027	Psoriasis	√	-	-	-	Extremities	53	F
PsO_028	Psoriasis	-	-	√	-	Palmoplantar	71	F
PsO_029	Psoriasis	-	-	√	-	Palmoplantar	47	M
PsO_030	Psoriasis	-	√	-	-	Extremities	22	M
PsO_031	Psoriasis	-	√	-	-	Trunk	30	F
PsO_032	Psoriasis	-	-	√	-	Palmoplantar	57	F
PsO_033	Psoriasis	-	√	√	-	Extremities	78	F
PsO_034	Psoriasis	-	-	√	-	Extremities	46	F
PsO_035	Psoriasis	-	√	√	-	Trunk	62	F
PsO_036	Psoriasis	-	-	√	-	Extremities	34	F
PsO_037	Psoriasis	-	-	√	-	Palmoplantar	27	M
PsO_038	Psoriasis	-	√	√	-	Extremities	80	M
PsO_039	Psoriasis	-	-	√	-	Trunk	56	M
PsO_040	Psoriasis	-	-	√	-	Trunk	35	M
PsO_041	Psoriasis	√	-	√	-	Trunk	34	M
PsO_042	Psoriasis	-	-	√	-	Extremities	71	F
PsO_043	Psoriasis	-	-	√	-	Extremities	50	F
PsO_044	Psoriasis	-	-	√	-	Trunk	74	M
PsO_045	Psoriasis	√	-	-	-	Extremities	38	F
PsO_046	Psoriasis	-	-	√	-	Extremities	85	F
PsO_047	Psoriasis	-	-	-	-	Extremities	46	F
PsO_048	Psoriasis	-	-	-	-	Head and neck	46	F
PsO_049	Psoriasis	-	-	-	-	Intertriginous	89	F
PsO_050	Psoriasis	-	-	-	-	Intertriginous	21	F
PsO_051	Psoriasis	-	-	-	-	Palmoplantar	70	F
PsO_052	Psoriasis	-	-	-	-	Palmoplantar	61	M
PsO_053	Psoriasis	-	-	-	-	Palmoplantar	65	F
PsO_054	Psoriasis	-	-	-	-	Palmoplantar	68	F
PsO_055	Psoriasis	-	-	√	-	Extremities	75	F
SW_001	Sweet's syndrome	-	√	-	-	Trunk	86	F
SW_002	Sweet's syndrome	-	√	-	-	Trunk	63	F
UR_001	undetermined rash	-	-	√	-	Extremities	75	F
UR_002	undetermined rash	-	-	-	-	Trunk	77	M
UR_003	undetermined rash	-	-	-	-	Extremities	80	F
UR_004	undetermined rash	-	√	√	-	Trunk	85	M
UR_005	undetermined rash	-	-	-	-	Extremities	23	F
UR_006	undetermined rash	-	-	-	-	Extremities	84	M
UR_007	undetermined rash	-	-	-	-	Extremities	65	M
UR_008	undetermined rash	-	-	-	-	Extremities	58	M
UR_009	undetermined rash	-	-	-	-	Trunk	61	M
UR_010	undetermined rash	-	-	-	-	Extremities	89	F
UR_011	undetermined rash	-	-	-	-	Trunk	69	M
UR_012	undetermined rash	-	-	-	-	Trunk	95	F
UR_013	undetermined rash	-	-	-	-	Trunk	32	M
UR_014	undetermined rash	-	-	-	-	Trunk	39	M
UR_015	undetermined rash	-	-	-	-	Trunk	20	F
UR_016	undetermined rash	-	-	-	-	Trunk	37	M
UR_017	undetermined rash	-	-	-	-	Extremities	39	M
UR_018	undetermined rash	-	-	-	-	Extremities	50	M
UR_019	undetermined rash	-	-	-	-	Extremities	92	F
UR_020	undetermined rash	-	-	-	-	Extremities	75	F
WELL_001	Well's syndrome	√	-	-	-	Extremities	54	M
WELL_002	Well's syndrome	√	-	-	-	Trunk	54	M
WELL_003	Well's syndrome	√	-	-	-	Intertriginous	50	M

Supplementary Table 5. Baseline characteristics of profiled patients

characteristic	value (N=270)
Age - median yr (range)	58 (19-95)
Female gender - %	35
Diagnosis - no.	
Psoriasis	55
Atopic Dermatitis	58
Lichen planus	18
Cutaneous lupus	19
Well's syndrome	3
Bullous pemphigoid	17
Drug hypersensitivity reaction	10
Hidradenitis suppurativa	5
Pyoderma gangrenosum	5
Sweet's syndrome	2
Dissecans cellulitis	3
Erythrodermas	30
Unclear rashes	20
Healthy skin	8
Location of biopsy - %	
Trunk	32.2
Extremities	51.5
Palmoplantar	4.9
Intertriginous	2.3
Head & Neck	7.6
Oral	1.1
Genital	0.4
